# Glioblastoma cells utilize evolutionarily adapted cell metabolism to promote their malignant proliferation

**DOI:** 10.1186/s40478-026-02318-7

**Published:** 2026-05-13

**Authors:** Maxim M. Bespalov, Vasiliki Gkini, Zeynep Iloglu, Seiya Yamada, Akira Nemoto, Pauliina Filppu, Kalevi Trontti, Liliia Andriichuk, Olli Pietiläinen, Vadim Le Joncour, Anni I. Nieminen, Pirjo Laakkonen, Takashi Namba

**Affiliations:** 1 Neuroscience Center, HiLIFE—Helsinki Institute of Life Science, University of Helsinki, Helsinki, Finland; 2 Division of Microscopic Anatomy, Graduate School of Medical and Dental Sciences, Niigata University, Niigata, Japan; 3 Translational Cancer Medicine Research Program, Faculty of Medicine, University of Helsinki, Helsinki, Finland; 4 ICAN Digital Precision Medicine Flagship Program, University of Helsinki, Helsinki, Finland; 5 Stem Cell and Metabolism Research Program, Faculty of Medicine, University of Helsinki, Helsinki, Finland; 6 Laboratory Animal Center, HiLIFE—Helsinki Institute of Life Science, University of Helsinki, Helsinki, Finland; 7 Department of Developmental Biology, Fujita Health University School of Medicine, Toyoake, Japan; 8 Division of Developmental Neurobiology, International Center for Brain Science (ICBS), Fujita Health University, Toyoake, Japan

**Keywords:** Glioblastoma, ARHGAP11B, Glutaminolysis, GOT2, Glutamic-oxaloacetic transaminase, Nucleotide biosynthesis, Mitochondrial metabolism, Metabolic reprogramming, Oncometabolism, Cell proliferation

## Abstract

Glioblastoma is the most aggressive primary brain tumor in adults, with limited therapeutic success and, therefore, poor prognosis. Its malignancy is partly driven by the high proliferative capacity of glioblastoma cells, yet the underlying molecular mechanisms remain unclear. Recent studies have revealed transcriptomic similarities between glioblastoma cells and human fetal neural stem/progenitor cells (NSCs), suggesting that glioblastoma may exploit developmental programs that promote NSC proliferation. Fetal human NSCs rely on glutaminolysis—a metabolic pathway induced by the human-specific mitochondrial protein ARHGAP11B—to sustain proliferation. Here, we show that *ARHGAP11B* expression correlates with glioma malignancy and is essential for glioblastoma cell proliferation, implicating a critical role of glutaminolysis in tumor growth. Among glutaminolysis-related enzymes, glutamic-oxaloacetic transaminase 2 (*GOT2*) shows a strong positive correlation with glioma grade and poor patient prognosis. Functional assays reveal that GOT2 knockdown significantly suppresses glioblastoma cell growth, indicating that GOT2-mediated glutaminolysis is critical for their proliferation. Metabolomic profiling further shows that GOT2 is required for nucleotide precursor synthesis, underscoring its role in supporting DNA replication. Consistently, GOT2 depletion reduces the proportion of glioblastoma cells in the S phase of the cell cycle. These findings suggest glioblastoma cells hijack an evolutionarily adapted metabolic program to support malignant growth.

**Supplementary Information:**

The online version contains supplementary material available at 10.1186/s40478-026-02318-7.

## Introduction

Glioblastoma is the most malignant brain tumor in adults with World Health Organization (WHO) grade 4 [1]. Patients with glioblastoma have very poor prognosis with an average life expectancy of only about one year after diagnosis [1]. Such a poor prognosis is mainly due to the high tumor cell adaptability enabling chemoresistance, and high proliferative capacity. Since higher frequency in mitotic figures is one of the histopathological prerequisites in diagnoses [2, 3], understanding the molecular mechanisms of glioblastoma’s extremely high proliferative capacity will provide important information for developing novel therapeutic strategies.

Recent advances in glioblastoma profiling shed light on the similarity between glioblastomas and neural stem/progenitor cells (NSCs) in the developing human neocortex [4, 5]. These two distinct cell types, one being a transformed malignant cell and the other one being a physiologically healthy cell, share key features such as high proliferative capacity and transcriptomic profiles [4, 5], suggesting that there is common machinery underlying glioblastoma’s and human fetal NSC’s high proliferation. Therefore, NSCs could provide a fundamental clue to understanding the aggressive proliferative capacity of glioblastomas, and thus their malignancy. One key aspect that determines the high proliferative capacity of human fetal NSCs is human evolution [6–9]. Human fetal NSCs are thought to have acquired mechanisms that drive the high proliferative capacity during the evolution toward *Homo sapiens* [6–9]. One of such evolutionarily adapted mechanisms is cell metabolism [10–12].

High proliferative cells, including tumor cells and NSCs, need to exhibit characteristic cell metabolism to supply essential metabolites to maintain their proliferative activity [13–18]. Of several metabolic pathways, glutaminolysis is shown to be characteristically upregulated in human fetal NSCs by a human-specific mitochondrial protein ARHGAP11B [19]. ARHGAP11B inhibits mitochondrial permeability transition pore formation or opening, thereby increasing Ca^2+^ concentration in the mitochondrial matrix, which leads to promotion of glutaminolysis [19]. Glutaminolysis is a pathway that converts glutamine to glutamate and then to alpha-ketoglutarate (αKG), which fuels the tricarboxylic acid (TCA) cycle. In light of this notion, here we hypothesize that the human evolution-associated cell metabolism, glutaminolysis, plays an important role in glioblastoma cell proliferation. While glioblastoma cells are known to utilize glutamine [18, 20], the mechanisms by which glutaminolysis controls the proliferative capacity of glioblastoma have not been elucidated well.

In the present study, we particularly focused on the last step of glutaminolysis, that is, glutamate to αKG conversion, and examined the mechanisms by which it regulates the proliferative capacity of glioblastoma cells. We found that glutamic-oxaloacetic transaminase 2 (GOT2)-dependent glutaminolysis and subsequent synthesis of nucleotide precursors are crucial for glioblastoma cell proliferation. Furthermore, a human-specific glutaminolysis inducer ARHGAP11B, whose role has been identified in the human NSCs [19, 21], also plays an important role in glioblastoma cell proliferation. These results shed light on the commonality in cell metabolism between glioblastomas and NSCs, which is potentially a target for the development of novel anti-tumor drugs.

## Materials and methods

### Glioblastoma cell lines

The BT12, BT13 and BT18 glioblastoma cell lines were originally obtained from glioblastoma patients by Prof. Pirjo Laakkonen [22, 23]. ZH305 cells were from University Hospital and University of Zurich [24].

BT12, BT13 and BT18 cells were cultured in Dulbecco’s Modified Eagle’s Medium/Nutrient Mixture F-12 Ham (11530566; Merk) supplemented with 2% B27 (11530536; Thermo Fisher Scientific), 1% glutamine, 15mM HEPES buffer (Lonza), 100 μg/mL streptomycin, epidermal growth factor (EGF, 20 ng/ml; AF-100-15-500UG; PeproTech), and basic fibroblast growth factor (bFGF, 10 ng/ml; AF-100-18B-250UG; PeproTech). ZH305 were cultured in Neurobasal medium supplemented with 2% B27, 1% glutamine, 15mM HEPES buffer (Lonza), 100 μg/mL streptomycin, EGF (20 ng/ml), and bFGF (20 ng/ml). For passaging cells were dissociated with Accutase (25-058-CI; Corning).

## Antibodies

Antibodies used in this study were as follows; anti-ARHGAP11B (mouse IgG1, 3758-A37-5, MPI-CBG, 1:200) [19], anti-HA (rabbit IgG, Cell Signaling Technology, #3724S, 1:2000 for immunocytochemistry, 1:5000 for immunoblot), anti-TOM20 (mouse IgG, Abcam, ab56783, 1:200 for OT2 (rabbit IgG, Cell Signaling Technology, #71692, 1:1000), anti-Ki67 (rat IgG2a, 14-5698-82, Thermo Fisher Scientific, 1:200), anti-GFP (chicken IgY, GFP-1020, Aves, 1:500), anti-α-Tubulin (mouse IgG, sc-5286, Santa Cruz Biotechnology, 1:5000),

anti-rat Cy3 (donkey IgG, 712-165-153, Jackson ImmunoResearch, 1:200), anti-chicken IgY Alexa Fluor 488 (donkey IgG, 703-545-155, Jackson ImmunoResearch, 1:500), anti-mouse Cy3 (donkey IgG, 715-165-151, Jackson ImmunoResearch, 1:200), anti-rabbit IgG Alexa Fluor 647 (donkey IgG, 711-605-152, Jackson ImmunoResearch, 1:500), anti-mouse IgG-horseradish peroxidase (HRP) (donkey IgG, 715-035-151, Jackson ImmunoResearch), anti-rabbit IgG-HRP (donkey IgG, 711-035-152, Jackson ImmunoResearch). The dilution of all HRP-labeled antibodies was 1:5000.

Anti-ARHGAP11B antibody was produced using humanARHGAP11B-specific peptide (CKALKKVNMKLLVNIREREDNV) in rabbit by Innovagen AB, Sweden. The specificity of protein G-affinity purified rabbit polyclonal anti-ARHGAP11B (16870.11) was tested by immunoblot (1:5000) of cell lysate in which ARHGAP11A or ARHGAP11B were overexpressed (see below; Fig. S1C).

## Mitochondrial fractionation and immunoblot

The mitochondrial fraction was obtained as previously described with some modifications [25, 26]. Glioblastoma cells were collected and washed twice with PBS at 4 °C. The cells were then suspended in fractionation buffer containing 10 mM HEPES (pH 7.4), 220 mM mannitol, 70 mM sucrose, and a protease inhibitor cocktail (Complete Mini, Merck Millipore), and homogenized with 10 strokes using a syringe with a 27-gauge needle. The crude postnuclear supernatant was obtained after two centrifugation steps at 800 × g for 5 min at 4 °C. It was then further centrifuged twice at 7,000 × g for 10 min each, followed by a final centrifugation at 10,000 × g for 10 min at 4 °C. The pellet was collected as the mitochondrial fraction. Equal amounts of protein from the total lysate and the mitochondrial fraction were used for immunoblotting (see below).

## Immunocytochemistry

For immunocytochemistry, dissociated glioblastoma cells were fixed with 4% paraformaldehyde (PFA) for 10 min and then washed with Milli-Q water once. The cells were resuspended in 200 µl of Milli-Q and 20 µl were added on a microscope slide, which was placed on heating plate at 50 °C to evaporate the liquid. Before the staining the cells were pre-treated with 0.01 M citrate buffer at 70 °C for 60 min and then left to cool down at room temperature (r.t.) for 20 min, followed by a phosphate buffered saline (PBS) wash. For permeabilization the cells were then incubated with 1% TritonX-100 in PBS for 30 min at r.t., followed by a PBS wash. Then any residual PFA was quenched with 0.1 M glycine in PBS for 30 min in r.t., followed by a PBS wash. Cells were left incubating with the relevant combination of the the primary antibodies in 1% bovine serum albumin (BSA) (mouse anti-ARHGAP11B, 1:500 or rabbit anti-ARHGAP11B, 1:500; rabbit anti-HA, 1:2000; rabbit or mouse anti-TOM20, 1:200; chicken anti-green fluorescent protein (GFP), 1:500) for 3 overnights at 4 °C. The cells were then washed 3 times with PBS and left incubating with the secondary antibodies (anti-mouse Cy3, 1:200 in 1% BSA; anti-rabbit Alexa 647, 1:500 in 1% BSA; anti-chicken Alexa 488, 1:500 in 1% BSA) and DAPI (1:1000) (D9542; Merk) at r.t. for 1 h. The cells were then washed 3 times with PBS and mounted a coverslip on top using Mowiol (475904-M, Merk). The cells were imaged using LSM980 (Zeiss) and ZEN software (Zeiss).

## Chemical compounds

iGOT (N-(4-Chlorophenyl)-4-(1 H-indol-4-yl)piperazine-1-carboxamide) was from BLDpharm (BD01294031). BPTES (Bis-2-(5-phenylacetamido-1,3,4-thiadiazol-2-yl)ethyl sulfide; 5.30030), CB-839 (N-(6-(4-(5-((2-Pyridin-2-ylacetyl)amino)-1,3,4-thiadiazol-2-yl)butyl)pyridazin-3-yl)-2-(3-(trifluoromethoxy)phenyl)acetamide; 5337170001) and R162 (2-Allyl-1-hydroxy-9,10-anthraquinone; 5380980001) were from Merk. All compounds were tested at 2.5–150 µM range (2.5, 5, 10, 25, 50, 100, and 150 µM). Calculation of IC50 was done by drc package (3.0–1) for R4.3.3.

### Plasmids

The shRNA targeting GLS (VB900044-4899hbn), GOT1 (VB900044-3426mfj), GOT2 (VB900044-3500fya), GOT2 version 2 (VB900044-3510sez) and scrambled shRNA (VB010000-0009mxc) were cloned into pLV-EGFP-T2A-Puro-U6 by VectorBuilder. ARHGAP11B-DN (previously called as ARHGAP11A220) [19], which was already cloned into pCR-Blunt II-TOPO, was subcloned into pCDH-CMV-T2A-EGFP. pCAGGS-empty [27], pCAGGS-ARHGAP11B-HA [19] and pCAGGS-ARHGAP11A-HA [19] were previously generated .

The shRNA-resistant GOT2 clone was synthesized by GeneArt (Thermo Fisher) and subcloned to pLex_307 by the Genome Biology Unit (GBU, University of Helsinki). The control LacZ gene was cloned from ORFeome collection into pLex_307 by GBU.

## Lentivirus production

The lentivirus was produced by Biomedicum Virus Core (HelVi-BVC) by co-transfecting a transfer plasmid (see above) together with a packaging plasmid psPAX2 and an envelope plasmid pMD2G into HEK293FT cells that were 50–70% confluent. Lenti Concentrator (Origene) was used to precipitate the viral particles and the pelleted viruses are resuspended in sterile 1x PBS (Gibco).

## Cells transduction, transfection and immunoblot

Glioblastoma cells were transduced with lentiviral particles made from constructs listed above by HelVi-BVC at 6.8 × 10 ^5^ pg/ml (p24 measurement). The average transduction efficiencies of the lentiviral vectors were more than 90% (GFP:95.4% ±2.01, ARHGAP11B-DN: 96.2% ±4.42, shScr: 97.5% ±1.06, shGOT2: 97.0% ±3.12, as mean ± SD).

For the transfection of 293T and glioblastoma cells up to 3 µg of plasmid per 1 million cells was used in mixture with Lipofectamine 2000 (Thermo Fisher Scientific). In experiments with shRNA-resistant GOT2 mutant cells were treated with 2 µg/ml puromycin (Thermo Fisher Scientific) starting 24 h post transfection for another 24 h prior to transduction with lentiviruses carrying shRNA.

For transfection of COS7 cells with pCAGGS-empty, pCAGGS-ARHGAP11A-HA or pCAGGS-ARHGAP11B-HA, cells were transfected with the plasmids using Lipofectamine 2000, and collected and lysed by 1xSDS sample buffer for immunoblot.

Total cell lysates were made 3 days after transduction or transfection, and processed for immunoblot. SDS-PAGE was performed using Novex Bis-Tris Gels (10%; ThermoFisher Scientific) in NuPAGE MOPS SDS RunningBuffer (ThermoFisher Scientific) according to the manufacturer’s protocol. After the electrophoresis, proteins were transferred onto PVDF membrane (Immobilon-P, Merck) in NuPAGE Transfer Buffer (ThermoFisher Scientific) for 2 h. The membranes were then incubated in TBST (Tris-buffered saline containing 0.1% Tween 20) containing 5% BSA for 1 h at r.t. with gentle shaking, followed by incubation with the indicated primary antibodies in TBST overnight at 4 °C with gentle shaking. The membranes were washed with TBST and then incubated with appropriate HRP-conjugated secondary antibodies in TBST for 1 h at r.t. with gentle shaking. Finally, membranes were developed with Pierce ECL Western Blotting Substrate (ThermoFisher Scientific). Exposure time was varied for best visibility. Images were acquired using PG-BOX Chemi XX6 (SYNGENE). The original immunoblot images are found in Supplementary File 1.

### Oxygen consumption rate (OCR) measurement

Oxygen consumption rate (OCR) measurements were conducted using a Seahorse XF96 Extracellular Flux Analyzer (Agilent Technologies), following a previously published protocol with minor modifications. BT13 cells were plated two days after transduction onto poly D lysine–coated XF96 cell culture plates (Agilent Technologies) at a density of 50,000 cells per well and maintained in growth medium for 20–24 h prior to analysis. OCR measurements were performed in Seahorse XF medium under a sequential injection protocol. Baseline OCR was recorded without added substrates, followed by the stepwise addition of: (i) 10 mM glucose, 10 mM galactose, and/or 2 mM glutamine; (ii) 2 µM oligomycin (75351, Merck); (iii) 100 µM 2,4 dinitrophenol (DNP; 34334, Sigma); and (iv) a combination of 1 µM antimycin A (A8674, Merck) and 1 µM rotenone (CRM38703, Merck) (A + R). All assays were conducted at 37 °C under atmospheric conditions without supplemental CO₂. Each experimental condition was measured in quadruplicate. Basal mitochondrial OCR (before substrate addition), substrate stimulated mitochondrial OCR (after substrate addition), and maximal mitochondrial OCR (calculated as OCR following oligomycin plus DNP treatment minus OCR after A + R addition) were determined by averaging three consecutive time points for each condition.

### Glioblastoma cell growth assay and cytotoxicity assay

Glioblastoma cell growth assay and cytotoxicity assay were performed using CellTiterGlo assay (Promega) and CellTox Green assay (Promega), respectively, according to manufacturer’s instructions. Briefly, cells were dissociated using Accutase (see above) and plated at 2500 cells/well density into 96-well plates. For experiments involving transduction, the dissociated cells were infected immediately, and the CellTiterGlo reagent was added directly to the wells at 1:1 ratio at 72 h or 7 days post-transduction. The data were from experiments using cells at 72 h post-transduction, otherwise specified. For experiments involving chemical compounds, the cells were allowed to recover for 24 h after the dissociation, then treated with the indicated compounds for 48 h. Subsequently, the CellTiterGlo reagent was added directly to the wells at 1:1 ratio, or CellTox Green reagent was added to the wells at 1:3000 final dilution. Cells were incubated the the CellTiterGlo reagent at RT for 10 min, or CellTox Green reagent for 20 min, and analyzed by FLUOstar plate reader (BMG Labtech). Alternatively, the biomass was measured 72 h post transduction of 1 million cells in a 35 mm dish, following a wash with PBS and lysis in 2x RIPA buffer using Pierce BCA Assay Kit (Thermo Fisher Scientific) according to manufacturer’s instructions and measuring absorbance at 562 nM using Multiskan FC (Thermo Scientific).

### Flow cytometry

The percentage of cells in S-phase was additionally assayed using Click-iT™ Plus EdU Alexa Fluor™ 647 Flow Cytometry Assay Kit (Thermo Fisher Scientific) according to manufacturer’s instruction. Briefly, at 72 h post-transduction, cells were treated with 10 µM EdU for 30 min followed by the Accutase treatment and subsequent fixation. Immediately after fixation with 4% PFA for 15 min cells were permeabilized with 0.1% TritonX/1% BSA in PBS for 15 min. The EdU labelling reaction took 30 min at RT on a rotator in darkness. This was followed by DAPI staining for 30 min and analyzed at Novocyte Quanteon 4025 (Flow Cytometry Unit, University of Helsinki).

For cell cycle analysis DAPI (D9542; Merk) staining was used. The glioblastoma cells were first treated with accutase then fixed for 15 min in 4% PFA at RT. Upon PBS washings cells were resuspended in 200 µl 1% BSA/PBS after addition of anti-GFP (1:500) and anti-Ki67 (1:500) antibodies the cells were rotated for 1 h at RT, then washed with PBS once and resuspended in 200 µl 1% BSA/PBS containing DAPI (1:1000), anti-chicken Alexa 488 (1:500) and anti-rat Alexa 647 (1:500) antibodies. Following 1 h RT incubation in dark on a rotator the cells were resuspended in 200 µl of PBS and analyzed at Novocyte Quanteon 4025 (Flow Cytometry Unit, University of Helsinki).

### Targeted metabolomics

At 3 days post-transduction with with shRNA-GOT2 or with shRNA-Scramble, BT13 cells were incubated with 500 µM ^13^C5, ^15^N1-glutamate (607851, Sigma) or ^13^C5, ^15^N2-glutamine (607983, Sigma) for 2 h at 37 °C in a humidified atmosphere of 5% CO2 for targeted metabolic flux analysis. Alternatively, BT13 cells at 4 days post-transduction with shRNA-GOT2 or with shRNA-Scramble were processed for targeted metabolomics. Cells were washed with ice-cold PBS, the metabolites were extracted from pelleted cells with 400 µl of cold extraction solvent (Acetonitrile: Methanol: Water; 40:40:20, Thermo Fischer Scientific) for targeted metabolomics and targeted metabolic flux analysis [17]. Subsequently, samples were vortexed three times for 2 min and sonicated for 1 min followed by centrifugation at 14,000 rpm at 4 °C for 5 min. Next the samples were centrifuged, the supernatant transferred to an evaporation tube and evaporated to dry under nitrogen stream. Samples were reconstituted in 40ul extraction buffer (40:40:20; Acetonitrile: Methanol: Water) and transferred to LC-MS vials. Supernatants were transferred into polypropylene tubes and placed into a Nitrogen gas evaporator. Dried samples were suspended with 40 µl of extraction solvent (Acetonitrile: Methanol: Water; 40:40:20) and vortexed for 2 min, then transferred into HPLC glass autosampler vials. 2 µl of sample were injected with Thermo Vanquish UHPLC coupled with Q-Exactive Orbitrap quadrupole mass spectrometer equipped with a heated electrospray ionization (H-ESI) source probe (Thermo Fischer Scientific). A SeQuant ZIC-pHILIC (2.1 × 100 mm, 5-µm particle) column (Merck) was used for chromatographic separation. The gradient elution was carried out with a flow rate of 0.100 ml/min with using 20 mM ammonium hydrogen carbonate, adjusted to pH 9.4 with ammonium solution (25%) as mobile phase A and acetonitrile as mobile phase B. The gradient elution was initiated from 20% Mobile phase A and 80% of mobile phase B and maintained till 2 min., followed by 20% Mobile phase A gradually increasing up to 80% till 17 min., then 80% to 20% Mobile phase A decrease in 17.1 min. and maintained up to 24 min. The column oven and auto-sampler temperatures were set to 40 ± 3 °C and 5 ± 3 °C, respectively. Following setting were used for MS: polarity switching; resolution of 35,000, the spray voltages: 4250 V for positive and 3250 V for negative mode; the sheath gas: 25 arbitrary units (AU); the auxiliary gas: 15 AU; sweep gas flow 0; capillary temperature: 275 °C; S-lens RF level: 50.0. Instrument control was operated with the Xcalibur software (Thermo Fischer Scientific). The peak integration was done with the TraceFinder 5.1 software (Thermo Fischer Scientific) using confirmed retention times standardized with library kit MSMLS-1EA (Merck). ^13^C isotopologues were analyzed with change of m/z (m + 1, m + 2 etc.). The data quality was monitored throughout the run using a pooled QC sample prepared by pooling 5 µL from each of the suspended samples and interspersed throughout the run as every 10th sample. The metabolite data was checked for peak quality, % relative standard deviation (RSD) and carryover. Each metabolite peak area was normalized to the total cell number analyzed and calculated as a ratio to the normalized peak areas of the relevant tracers, or as a fraction of the sum of all isotopologues. Isotopologues containing ^13^C and/or ^15^N are expressed as, for example, ^13^C2-aspartate, which indicates the labeled aspartate has two ^13^C regardless of their position.

### RNA sequencing and data analysis

Sample Preparation.

Total RNA was isolated from lysed cells, which were transduced with GFP-expressing lentivirus 7 days before the cell lysis, using the NucleoSpin RNA plus kit (740984, Macherey-Nagel) according to the manufacturer’s instructions. To ensure that no residual DNA was left, the samples were digested with rDNAse using the NucleoSpin rDNAse set (740963, Macherey-Nagel) followed by a repurification of the RNA using the RNA Clean-up XS kit (740903, Macherey-Nagel).

RNA-sequencing.

Library preparation from 200 ng of total RNA was performed according to NEBNext Ultra II Directional RNA Library Prep Kit for Illumina Instruction Manual, Sect.  1 - Protocol for use with NEBNext Poly(A) mRNA Magnetic Isolation Module (New England Biolabs, Ipswich, MA, USA). The number of PCR cycles was 10 in the library amplification step. Libraries were barcoded with NEBNext^®^ Multiplex Oligos for Illumina (New England Biolabs, Ipswich, MA, USA). Library quality check was performed using TapeStation High Sensitivity DNA ScreenTape analysis (Agilent, Santa Clara, Ca, USA) and the libraries were pooled based on the concentrations acquired from Quant-iT dsDNA high sensitivity assay (Thermo Fisher Scientific, Waltham, MA, USA). MiSeq Nano 2 × 150 bp sequencing was done for quality controlling purposes. Sequencing was performed with Illumina NovaSeq X 25B 200c flowcell lane (Illumina, San Diego, CA, USA. Read length for the paired-end run was 2 × 101 bp.

### Data analysis

RNA analysis up to raw gene counts was done using the Illumina Novaseq X on-board DRAGEN system (Illumina, San Diego, CA, USA; DRAGEN version 4.3.16, RNA app version 1.3.13; Reference GRCh38). Raw gene count data were processed in R (version 4.5.3) and were filtered to remove genes with counts per million (CPM) < 1 in fewer than 3 samples, TMM-normalized using edgeR (version 4.8.2) [28], and log2-CPM expression values were obtained using voomWithQualityWeights from the limma package (version 3.66) [29]. Gene annotation was performed via biomaRt (Ensembl GRCh38). Transcriptional subtype scoring was performed using the MES, AC, OPC, and NPC cellular state signatures from Neftel et al. [30]. Signature genes were z-score scaled across samples and visualized as a heatmap using the ComplexHeatmap (version 2.26.1) [31] package, with rows split by subtype and columns clustered by Pearson correlation. All data have been submitted to Gene Expression Omnibus (GSE329419; https://www.ncbi.nlm.nih.gov/geo/).

### Gene expression analysis using publicly available datasets

**Fig. 1:**
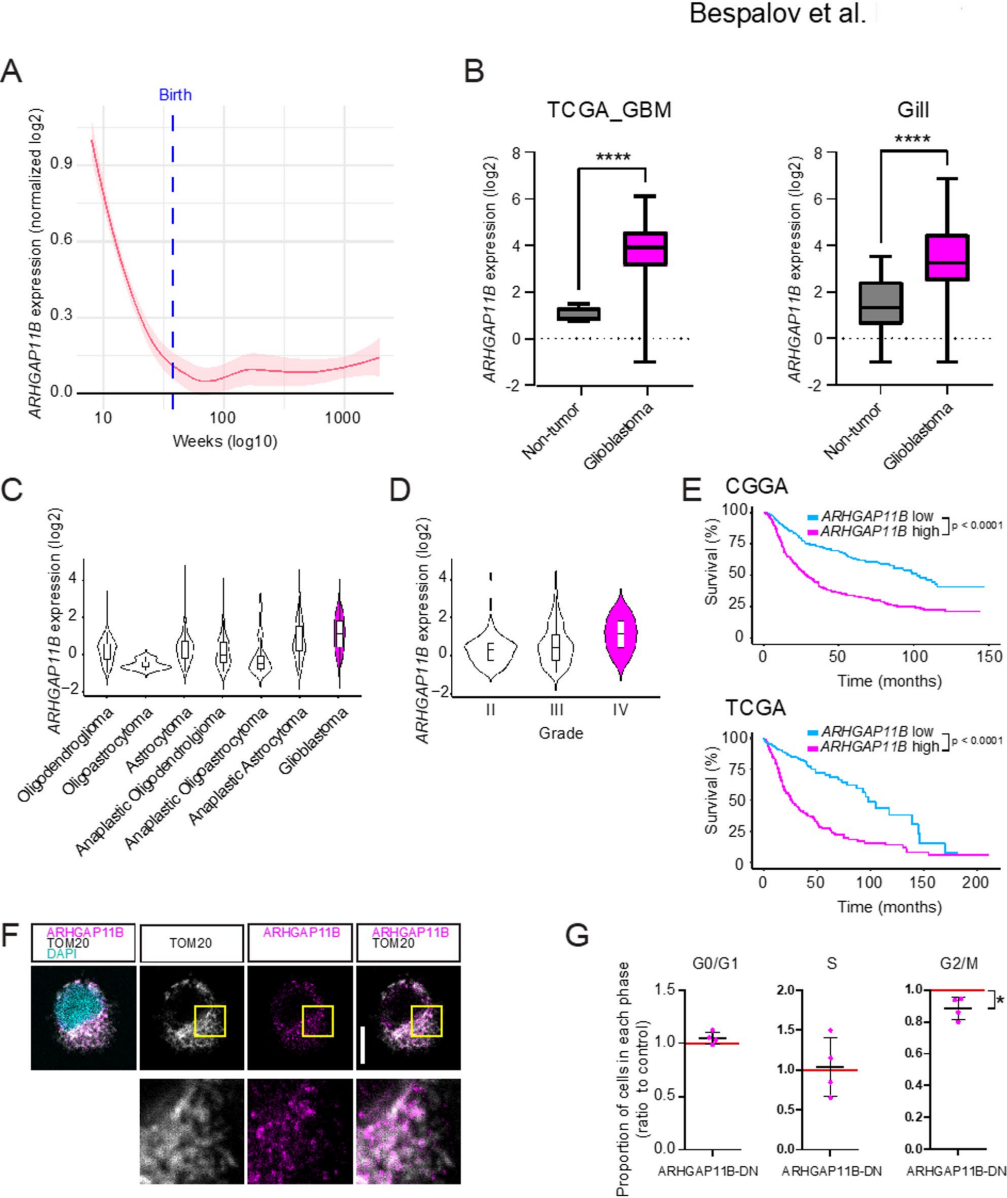
ARHGAP11B expression in glioblastoma cells. **A** *ARHGAP11B* expression in human brains from post-conceptional weeks 8 (embryo) to 1958 (40 years old). Reanalysis of BRAINSPAN transcriptome data [34]. A smoothed trend line (red) and a 95% confidence interval (light pink). Blue dashed line indicates the week of birth. **B** *ARHGAP11B* expression in non-tumor tissues (gray) and glioblastoma tissues (magenta). Reanalysis of transcriptome data from TCGA_GBM [35] and Gill et al. [36] using GlioVIs [37]. ****p < 0.0001, Mann-Whiteny test for TCGA_GBM; ****p < 0.0001, *t* = 6.154, df = 31.84, Welch’s t test for Gill. **C**-**E** CGGA (ref) data on *ARHGAP11B* expression in different glioma types (**C**) and grades (**D**), and association between the expression and patient survival (**E**). *p*-value by a log-rank test (Mantel–Cox non-parametric comparison). Reanalysis of transcriptome data from CGGA [38] and TCGA datasets using GlioVis. **F** Immunofluorescence of BT13 glioblastoma cell for TOM20 (white), ARHGAP11B (magenta; mouse antibody) with DAPI staining (cyan). Boxed areas in the upper panels are shown at higher magnification on the bottom. Scale bar: 10 μm. **G** Quantification of the proportion of cells in each phase of the cell cycle upon ectopic expression of dominant negative ARHGAP11B (ARHGAP11B-DN). BT13 glioblastoma cells were transduced with lentivirus carrying ARHGAP11B-DN and the cell cycle was analyzed by flow cytometry based on DAPI signal intensity. The proportion is expressed as a ratio of ARHGAP11B-DN transduced cells to control, which is GFP-transduced cells. The red line at 1.00 indicates the ratio obtained if ARHGAP11B-DN transduction would have no effect. *p = 0.046, *t* = 3.297, df = 3, one sample t test. Error bars, SD

**Fig. 2:**
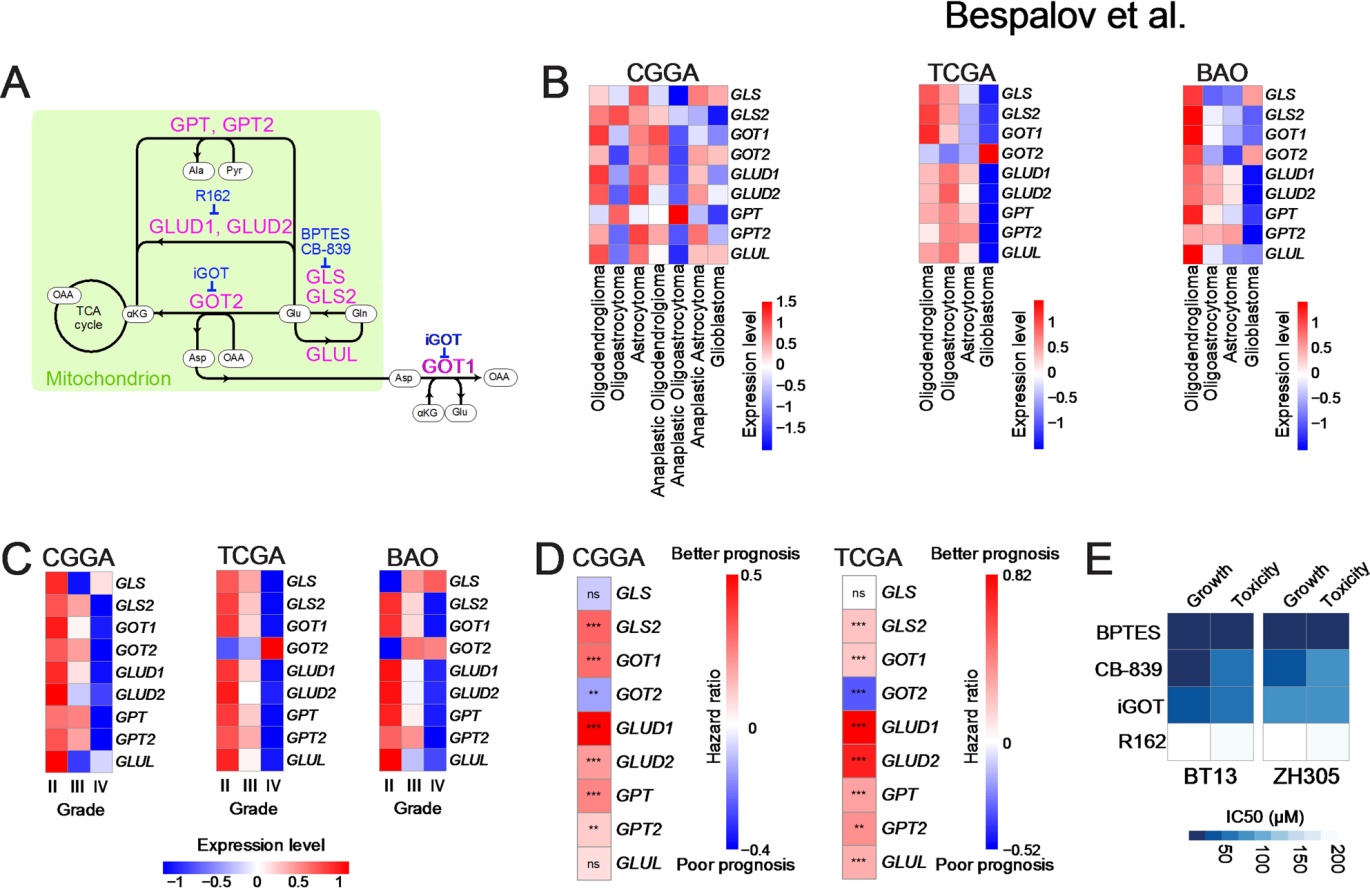
Expression of glutaminolysis regulatory genes in glioblastoma cells. **A**Schematic illustration of glutaminolysis and the enzymes that regulate it(magenta). Specific inhibitors to the enzymes (blue).**B**-**D** Reanalysis of transcriptomedata from CGGA [34,63],TCGA [38],and Bao et al. [37]Expression of genes encoding glutaminolysis regulatory enzymes in differentglioma types (**B**) and grades (**C**), and association between the geneexpression and patient prognosis expressed as hazard ratios (**D**).Significance for log_10_-transformed hazard ratios was assigned basedon Wilcoxon test with FDR correction. ns: *p* ≥ 0.05; 0.001 ≤ **p< 0.01; ***p < 0.001. (**E**) Quantification of IC50 in μM ofinhibitors against glutaminase (BPTES and CB-839), glutamic-oxaloacetictransaminases (iGOT) and glutamate dehydrogenases (R162) on BT13 and ZH305glioblastoma cells. IC50 values were calculated based on the growth andcytotoxicity induced by the inhibitors. See also Supplementary data 1 for B-D.

All gene expression data were obtained from BRAINSPAN (https://www.brainspan.org/; Fig. 1A), GlioVis (https://gliovis.bioinfo.cnio.es/; Figs. 1B-E and 2B-D, Fig. S1A, B, Fig. S3A) and GTEx [32] using GEPIA2 (http://gepia2.cancer-pku.cn/; Fig. S5)[33]. The data were analyzed using Rstudio and GraphPad Prism.

### Statistics and plots

Statistical tests used in this study and relevant information are described in the figure legends. Normal distribution of data was tested by Shapiro-Wilk test. The IC50 calculations were performed using drc package (3.0–1) for R (4.3.3). Statistics and plots were made with GraphPad Prism (v10.5.0), Scatterplot.bar (v.0.7.0; https://scatterplot.bar), Rstudio (v.2025.05.1) and R (4.3.3). For the heatmaps on Fig. 4B and C first the original peak areas were normalized by total ion count then by the tracer (^13^C5, ^15^N1-glutamate or ^13^C5, ^15^N2-glutamine) itself. For the heatmap on Fig. 4E the original metabolites peak areas were normalized by total ion count. The significance for each pair shGOT/shSCR was determined by Welch’s two-sample t-test before the data were ratio-transformed. The corresponding p-values for each pair of isotopologues are shown in Supplementary Data 1.

## Results

### Human-specific glutaminolysis inducer ARHGAP11B is enriched in glioblastoma cells

To seek a contribution of evolutionarily adapted cell metabolism in glioblastoma cells, we first examined the expression of the key regulator of such metabolism, namely ARHGAP11B. Previous studies showed that *ARHGAP11B* is enriched in human fetal NSCs and the protein is localized in mitochondria to act as a glutaminolysis inducer [17, 19]. The expression of *ARHGAP11B* in the non-pathological human brain was the highest during the first trimester of development and gradually decreased by the end of the fetal period (Fig. 1A). Therefore, the expression in the adult brain was considerably lower than that in the fetal brain (Fig. 1A). However, there is a condition that exhibits exceptionally high expression of *ARHGAP11B* even in the adult brain, that is, glioblastoma. The expression of *ARHGAP11B* in the glioblastoma tissues was significantly higher than in non-tumor brain tissues (Fig. 1B), suggesting re-activation of a human fetal brain-like *ARHGAP11B* expression in the adult patients’ brain with glioblastoma. We next compared the expression of *ARHGAP11B* in different glioma subtypes and found that glioblastoma cells exhibited the highest expression of *ARHGAP11B* among the glioma subtypes analyzed (Chinese Glioma Genome Atlas (CGGA) dataset [39]; Fig. 1C). Consistent with the highest expression of *ARHGAP11B* in glioblastoma cells, significantly higher expression of *ARHGAP11B* was also detected in grade IV glioma cells (Fig. 1D) in the CGGA data set, which used the categorization guideline by WHO published in 2016 [40]. *ARHGAP11B* expression was observed in all three glioblastoma subtypes, that is, classical, mesenchymal-like (MES), and proneural (PN) (Fig. S1A). Notably, patients with glioma expressing *ARHGAP11B* at a relatively higher level showed worse prognosis compared to those with relatively lower expression of *ARHGAP11B* (Fig. 1E), suggesting a correlation between *ARHGAP11B* expression and tumor aggressiveness. To confirm the expression of ARHGAP11B at the protein level in glioblastoma cells, we first performed biochemical assay using four patient-derived glioblastoma cell lines (BT12, BT13, BT18, ZH305), which have been previously characterized [41], and further validated by RNA sequencing (Fig. S2). BT12 and BT13 have previously been classified as MES subtype glioblastoma cells, whereas BT18 and ZH305 have been classified as PN subtype cells [42]. In the present study, hierarchical clustering analysis showed that BT12 clustered closely with BT13, and BT18 clustered with ZH305, indicating high similarity within each pair (Fig. S2). Thus, although glioblastoma subtypes are increasingly recognized as existing along a continuum rather than as discrete categories [30, 43], the classification of glioblastoma cells used in this study is largely consistent with previous characterizations [44]. ARHGAP11B protein was enriched in the mitochondrial fraction (Fig. S1C, D) as same as the well-known mitochondrial protein TOM20 (Fig. S1D). To corroborate the biochemical analysis, we performed immunocytochemistry on BT13, and found that the immunofluorescence signal of ARHGAP11B was colocalized with TOM20 (Fig. 1F, Fig. S1E). These results showed that ARHGAP11B is expressed in the mitochondria of glioblastoma cells, similar to the human NSCs [19]. Interestingly, there were positive correlations between the expression of *ARHGAP11B* and classical NSC markers, that is, *CD44*,* NES*,* PROM1* and *SOX2* (Fig. S1B).

We next examined whether ARHGAP11B regulates glioblastoma cell metabolism and mitosis. To this end, we inhibited the function of ARHGAP11B in a patient-derived glioblastoma cell line, using a dominant negative (DN) form of ARHGAP11B [17, 19]. As previously shown [19], ARHGAP11B-DN was localized in mitochondria (Fig. S1F), where it is known to inhibit endogenous ARHGAP11B function. A previous study further demonstrated that ARHGAP11B expression enhances glutamine-dependent mitochondrial respiration [19]. We therefore examined whether inhibition of ARHGAP11B reduces glutamine-dependent mitochondrial respiration in glioblastoma cells. To this end, we measured the mitochondrial oxygen consumption rate (OCR) in glioblastoma cells expressing either GFP (control) or ARHGAP11B-DN, in the presence or absence of glutamine supplementation. Control glioblastoma cells showed a greater increase in OCR after glutamine addition compared to ARHGAP11B-DN-expressing cells, indicating that the control cells are capable of utilizing glutamine to support mitochondrial respiration more than cells with ARHGAP11B inhibition (Fig. S1G). To further corroborate this finding, we compared the maximal mitochondrial OCR between control and ARHGAP11B-DN–expressing cells under uncoupled conditions induced by 2,4-dinitrophenol (DNP). In control cells, maximal OCR was higher in the presence of glutamine than in its absence. In contrast, the glutamine-induced increase in maximal OCR was significantly attenuated in ARHGAP11B-DN–expressing cells compared with control cells (Fig. S1H). Together, these results indicate that ARHGAP11B promotes glutaminolysis and supports glutamine-dependent mitochondrial respiration in glioblastoma cells, similar to the human NPCs [19].

We then examined the effect of ARHGAP11B inhibition on glioblastoma cell proliferation. The proportion of cells in each cell cycle phase, that is, the Gap0/Gap1 (G0/G1)-, the synthesis phase (S)- and the Gap 2/Mitosis (G2/M)-phases, was analyzed by flow cytometry. We found that the proportion of cells in the G2/M-phase was significantly reduced upon overexpression of ARHGAP11B-DN, compared to the control cells transduced with a GFP-expressing vector (Fig. 1G). The proportions of cells in the other cell cycle phases were not statistically changed (Fig. 1G). To further explore the changes in the G2/M-phase, the cells in the G2-phase and the M-phase were segregated based on Ki67 intensity, which is the highest in the cells during M-phase (Fig. S1I). While the proportion of cells in the M-phase was not changed, the proportion of cells in the G2-phase was significantly reduced upon ARHGAP11B-DN overexpression (Fig. S1J). In addition, no changes in the proportion of cells that underwent apoptosis, which is based on DNA contents, were observed upon ARHGAP11B-DN overexpression (GFP: 0.69%±0.11, ARHGAP11B-DN: 0.71%±0.16 as mean ± SD). These results suggest that the evolutionarily adapted glutaminolysis may play a role in glioblastoma cell growth.

### Glutaminolysis is crucial for glioblastoma cell growth

To examine whether glutaminolysis regulates glioblastoma cell growth, we first analyzed the expression of the key glutaminolysis regulatory genes (Fig. 2A) in three publicly available datasets [35, 39, 45]. Although the glutamine to glutamate conversion is mainly mediated by enzymes named glutaminases, encoded by *GLS* and *GLS2*, glutamate to αKG conversion could be catalyzed by three distinct enzymes, that is, glutamate dehydrogenases (encoded by *GLUD1* and *GLUD2*), glutamic-oxaloacetic transaminases (encoded by *GOT1* and *GOT2*) and glutamic-pyruvic transaminases (encoded by *GPT1* and *GPT2*) [46–48]. In addition, glutamate to glutamine conversion is catalyzed by glutamine synthetase encoded by *GLUL*. Of these genes, *GLS* and *GOT2* were highly expressed in glioblastoma cells (Fig. 2B), as well as in grade IV gliomas (Fig. 2C). Interestingly, *GOT2* was the only gene expression which was significantly associated with poor prognosis, while the expression of the other genes was significantly associated with better prognosis in at least one data set (*GLS2*,* GOT1*, *GLUD1*, *GLUD2*, *GPT*, *GPT2* and *GLUL*) or had no significant association with prognosis (*GLS*) (Fig. 2D). These results strongly suggest that, of three distinct glutaminolysis pathways, a pathway mediated by glutaminase (*GLS*) and glutamic-oxaloacetic transaminase (*GOT2*) is potentially involved in glioblastoma progression.

To investigate the functional relevance of these enzymes, the patient-derived glioblastoma cells were treated with compounds that inhibit glutaminases (BPTES and CB-839), and glutamic-oxaloacetic transaminases (iGOT) [49, 50] (Fig. 2E), followed by measurement of cell viability and cytotoxicity. As a negative control, we also inhibited glutamate dehydrogenases (GLUD1 and GLUD2) by R162, which are enzymes not expected to be involved in the glioblastoma growth according to the gene expression pattern (Fig. 2B-D). As previously shown in glioblastoma [18], inhibiting glutaminases (GLS and GLS2) significantly reduced cell viability and increased cytotoxicity (Fig. 2E). Interestingly, inhibition of glutamic-oxaloacetic transaminases significantly attenuated the growth and increased cytotoxicity (Fig. 2E), while no significant changes were observed upon inhibition of glutamate dehydrogenases, showing that the branch of glutaminolysis mediated by glutamic-oxaloacetic transaminases is functionally crucial for glioblastoma growth.

### Suppressing GOT2 expression causes growth reduction of glioblastoma cells

**Fig. 3:**
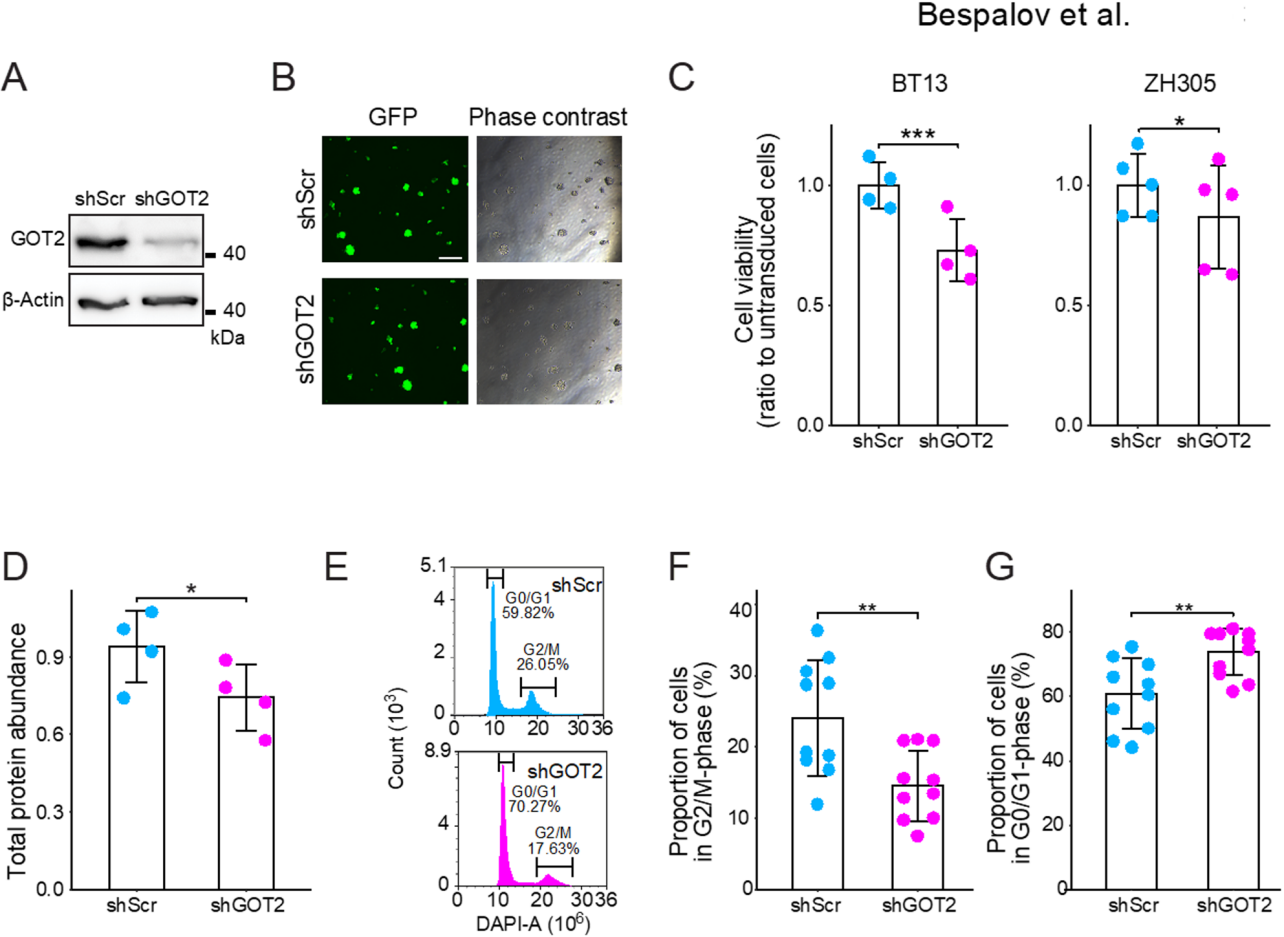
Knockdown of GOT2 suppresses glioblastoma growth. **A** Effects of shRNA against GOT2 (shGOT2) and control shRNA with a scrambled sequence (shScr) on the expression of the indicated proteins were examined by immunoblot using anti-GOT2 antibodies. β-actin was used as a loading control. **B** Fluorescent image of BT13 cells transduced with lentivirus carrying shScr or shGOT2 and GFP, together with phase contrast images. Scale bar: 200 μm. **C** Quantification of cell viability of BT13 (left panel) and ZH305 (right panel) glioblastoma cells transduced with lentivirus carrying shScr (cyan) or shGOT2 (magenta). The cell viability is expressed as a ratio of transduced cells to untransduced cells. Significance by paired t-test (BT13: ***p = 0.0009, t = 13.378, df = 3; ZH305: * *p* = 0.0357, t = 3.1165, df = 4). **D** Quantification of total protein abundance of cells transduced with lentivirus carrying shScr (cyan), or shGOT2 (magenta). The total protein abundance is expressed as a ratio of transduced cells to untransduced cells. Statistics by paired t-test: *p = 0.027, t = 4.0352, df = 3. **E**-**G** Cell cycle analysis of BT13 cells transduced with lentivirus carrying shScr (cyan), or shGOT2 (magenta). The cell cycle was analyzed by flow cytometry based on DAPI signal intensity. Flow cytometry plots of cells with indications of the cells in the G0/G1-phase and G2/M-phase (**E**; abscissa, DAPI area intensity (DAPI-A); ordinate, cell counts). Quantification of the proportion of cells in the G2/M-phase (**F**) and G0/G1-phase (**G**) of the cell cycle. Statistics by Student’s t-test (G0/G1: **p = 0.0063, t = -3.093, df = 18; G2/M-phase: **p = 0.0054, t = 3.159, df = 18). Error bars, SD

According to the gene expression patterns of *GOT1* and *GOT2* (Fig. 2B-D), GOT2 is likely to play a critical role in glioblastoma growth. Similar to ARHGAP11B, GOT2 protein was found to be enriched in the mitochondria in all four glioblastoma cell lines examined (Fig. S1D). In addition, *GOT2* expression was similar among distinct glioblastoma subtypes (Fig. S3A). Therefore, we examined the functional relevance of GOT2 in glioblastoma cell growth of BT12, BT13, BT18 and ZH305 cell lines by suppressing the expression of GOT2 using short hairpin RNA (shRNA)-mediated knockdown (KD). The KD efficiency was confirmed as the reduction in the endogenous GOT2 protein expression level (Fig. 3A). More than 90% of glioblastoma cells were transduced by the KD vector (Fig. 3B). Knockdown of GOT2 (shGOT2) significantly reduced the cell viability of all glioblastoma cell lines examined compared to control cells transduced with a scramble shRNA (shScr) both at 3 (BT13 and ZH305; Fig. 3C) and 7 (BT12, BT13, BT18 and ZH305; Fig. S3B) days after the transduction, consistent with the strong association of *GOT2* in glioblastoma malignancy (Fig. 2B–D). The reduction in the number of cells upon GOT2 KD was also reflected in the decreased abundance of total protein, which is a reliable proxy of total cell mass [51], at 3 days post-transduction (Fig. 3D). Furthermore, no changes in the proportion of cells that underwent apoptosis, which is based on DNA contents, were observed upon GOT2 KD (shScr: 4.167%±0.91, shGOT2: 2.64%±2.17 as mean ± SD). Interestingly, overexpression of GOT2 did not influence the cell viability of glioblastoma cells (Fig. S3C, D). These results suggest that GOT2 is required, but not sufficient, to promote glioblastoma cell growth.

To examine whether GOT2 is required for glioblastoma cell proliferation, the proportions of cells in the G2/M-phase and G0/G1-phase were analyzed by flow cytometry based on the signal intensity of 4’,6-diamidino-2-phenylindole (DAPI), a fluorescent dye that binds to double-stranded DNA, upon GOT2 KD in the patient-derived glioblastoma cells (Fig. 3E, Fig. S1G). The proportion of cells in the G2/M-phase was significantly reduced in the GOT2-KD cells (Fig. 3F), and reciprocally, the proportion of cells in the G0/G1-phase was significantly increased (Fig. 3G). We further examined the effect of GOT2 KD on G2- and M-phases separately by combining DAPI and Ki67 staining. The proportion of cells in G2- and M-phases was significantly reduced upon GOT2 KD (Fig. S3H, I). Taken together, these results indicate that GOT2 is required for cell proliferation, thus the growth of glioblastoma cells.

Finally, the specificity of KD was validated in two ways as previously described [52]: (i) Rescuing the KD phenotype by expression of shRNA-resistant target gene, and (ii) reproducing the phenotype by two different shRNA sequences. We first examined whether an ectopic expression of shRNA-resistant GOT2 rescues the reduction of cell growth. To this end, we transfected glioblastoma cells with the shRNA-resistant GOT2 and LacZ as negative control, and then transduced the cells with shRNA vectors. The expression of the shRNA-resistant GOT2 suppressed the effect of shGOT2 on glioblastoma cell growth (Fig. S3C, D), suggesting that the negative effect of shGOT2 on cell growth is specific to GOT2 silencing. In addition, we reproduced the effect of GOT2 KD by another shRNA, namely shGOT2-2, which has a distinct shRNA sequence (Fig. S3E, F). These results indicate that the shGOT2-induced growth suppression was actually caused by the KD of GOT2 expression.

#### GOT2 provides glioblastoma cells with essential nucleotide precursors for DNA synthesis

**Fig. 4:**
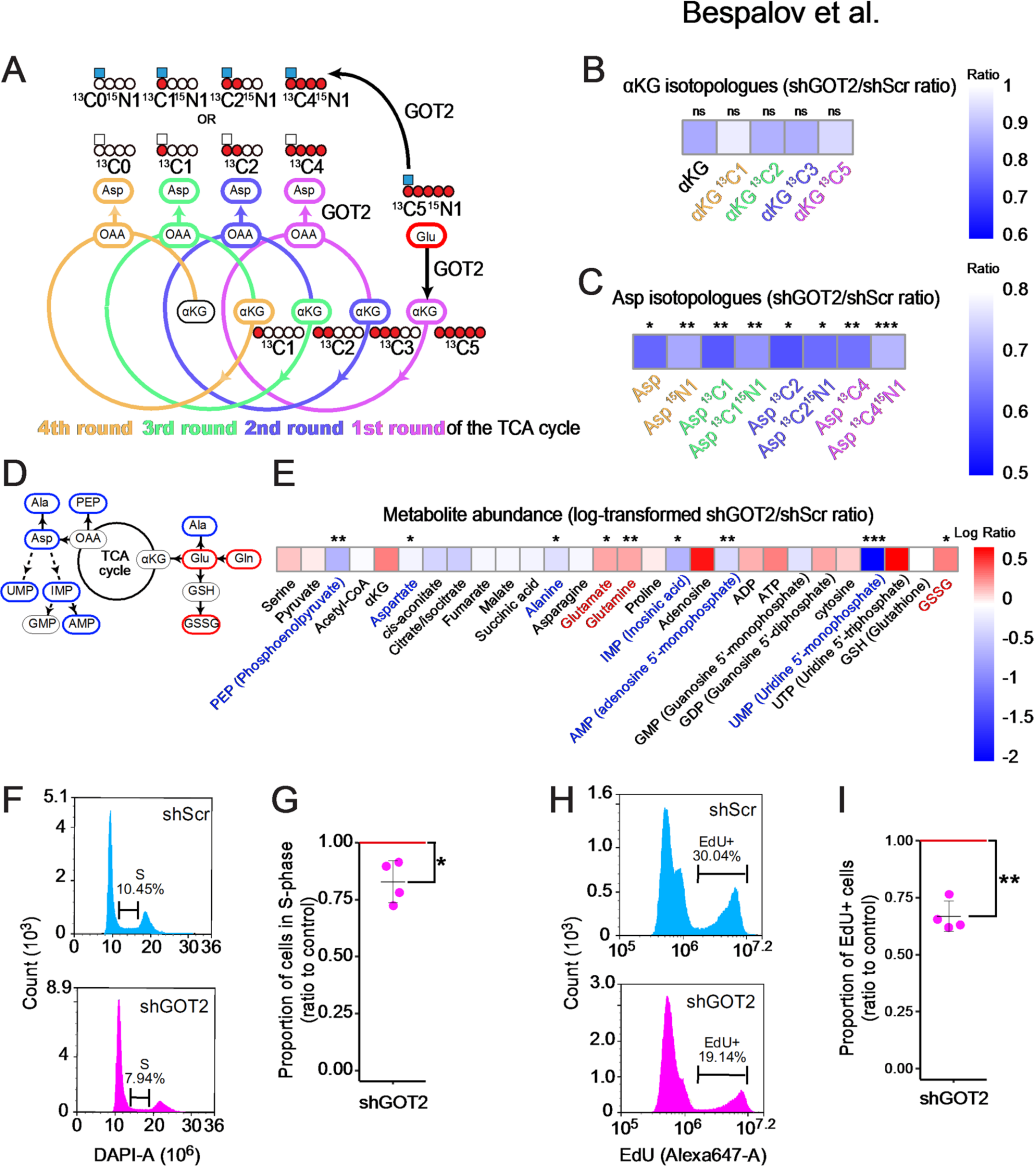
Knockdown of GOT2 decreases the production of aspartate and nucleotide precursors, and DNAsynthesis. **A** Schematic illustration of selected aspects of glutamate metabolism.C5,N1-glutamate (Glu) can be metabolized to alpha-ketoglutarate (αKG) and then aspartate (Asp) via oxaloacetate (OAA) through the TCA cycle. The first round of the TCA cycle, starting fromC5-αKG, producesC4-Asp and/orC4,N1-Asp (magenta). The second round of the TCA cycle producesC3-αKG andC2-Asp and/orC2,N1-Asp (blue). The third round of the TCA cycle producesC2-αKG andC1-Asp and/orC1,N1-Asp (green). The fourth round of theTCA cycle producesC1-αKG and Asp without isotopes and/orN1-Asp (orange).C andN atoms are indicated by red circles and bluesquares, respectively.C andN atoms are indicated by empty circles and squares, respectively.** B, C **BT13 cells were transduced by lentiviruscarrying shScr or shGOT2, and were incubated with 0.5mM ofC5,N1-glutamate (tracer) for 2 hours, followed by metabolome analysis.Heatmap of αKG **(B)** and Asp **(C)** isotopologue peak areas normalized byC5,N1-glutamate peak areas, each expressed as a ratio of shGOT2transduced cells to the control shScr-transduced cells. The original peak areas were first normalized by total ion count then by the peak areas ofthe tracer itself (Supplementary Data 1). Statistical significance was determined by Welch’s t-test. **D, E **BT13 cells were transduced by lentiviruscarrying shScr or shGOT2, followed by metabolome analysis. Summary of significantly increased (red) and decreased (blue) metabolites uponGOT2 knockdown **(D)**. Heatmap of 28 metabolites detected, each expressed as a natural-log-transformed ratio of shGOT2 transduced cells toshScr-transduced cells **(E)**. The original peak areas were normalized by total ion count (Supplementary Data 1). Statistical significance wasdetermined by Welch’s t-test.** F-I** BT13 cells were transduced by lentiviruses carrying shScr or shGOT2, and incubated with EdU for 30 min,followed by flow cytometric analysis. Flow cytometry plots of cells with indications of the cells in the S-phase (**F**: abscissa, DAPI area intensity(DAPI-A); ordinate, cell counts). Quantification of the proportion of cells in the S-phase of the cell cycle upon GOT2 knockdown **(G)**. Statisticalsignificance was determined by one-sample t-test. *p= 0.0382, t = -3.5447, df = 3. Flow cytometry plots of EdU+ cells (**H**: abscissa, Alexa647 areaintensity (Alexa647-A) as EdU+ signal intensity; ordinate, cell counts). Quantification of the proportion of EdU+ cells upon GOT2 knockdown **(I)**.Statistical significance was determined by one-sample t-test. **p=0.0010, t = -12.78, df = 3. Error bars, SD. See also Supplementary data 1 for B,C, E.

GOT2 is known to catalyze the conversion of glutamate and oxaloacetate (OAA) to αKG and aspartate, thus, the decreased GOT2 expression would consequently reduce the amount of αKG and aspartate. Therefore, we investigated the effect of GOT2 KD on the pathways that produce αKG and aspartate from glutamate, a substrate of GOT2, in glioblastoma cells. To this end, the glioblastoma cells transduced with shGOT2 or shScr were incubated with a metabolic tracer, ^13^C5, ^15^N1-glutamate, which consists of five ^13^C and one ^15^N, for 2 h. After the incubation, cells were processed for metabolic flux analysis, by which derivatives of the tracer containing ^13^C and ^15^N were detected. The incorporation of ^13^C5, ^15^N1-glutamate was not changed upon GOT2 KD (Fig. S4A). We first quantified all isotopologues of aspartate containing at least one ^13^C or ^15^N (Fig. 4A). Since the nitrogen atom of glutamate is transferred to OAA to generate aspartate by GOT2, the ^15^N in aspartate is likely a product of transamination of the ^15^N of glutamate to OAA. Glutamate is converted into αKG (^13^C5) and then fuels the first round of the TCA cycle (Fig. 4A, magenta), which produces aspartate (^13^C4) through OAA. A portion of ^13^C enters the second round of the TCA cycle to produce αKG (^13^C3) and aspartate (^13^C2) (Fig. 4A, blue), then the third round of the cycle producing αKG (^13^C2) and aspartate (^13^C1) (Fig. 4A, green), and finally the fourth round of the cycle, which generates αKG (^13^C1) (Fig. 4A, orange). After the fourth round, ^13^C is no longer detected in the aspartate.

To analyze the effect of GOT2 KD on the production of αKG and aspartate from glutamate, we compared the proportion of each isotopologue to the tracer, ^13^C5, ^15^N1-glutamate, in the GOT2 KD cells with the control cells. If the amount is decreased upon GOT2 KD, the ratio of shGOT2 to shScr is lower than 1. No significant changes were detected in the shGOT2/shScr ratio of αKG containing at least one ^13^C, which is derived from ^13^C5, ^15^N1-glutamate (Fig. 4B). Absence of significant reduction in αKG production from glutamate suggests glioblastoma cell adaptability, which utilizes alternative metabolic pathways producing αKG to compensate the KD effect of GOT2. In contrast, the shGOT2/shScr ratio of aspartate, which contains at least one ^13^C and/or ^15^N, was significantly reduced (Fig. 4C), suggesting that the activity of pathways producing aspartate from glutamate is attenuated upon GOT2 KD. In addition to this pathway, a part of aspartate can be produced from glutamate through reductive carboxylation [53]. Through this reaction ^13^C5, ^15^N1-glutamate could end up ^13^C3, ^15^N1- or ^13^C3-aspartate. The amount of ^13^C3, ^15^N1- or ^13^C3-aspartate was approximately half of the amount of ^13^C4, ^15^N1- or ^13^C4-aspartate (Fig. S4B, Supplementary data 1). This result suggests that the contribution of the reductive carboxylation to the production of aspartate is limited, although the amount of ^13^C3, ^15^N1- or ^13^C3-aspartate was also decreased significantly upon GOT2 KD (Fig. S4B, Supplementary data 1). These results indicate that glioblastoma cells mainly utilize GOT2 to generate aspartate, in which carbon atoms are largely derived from glutamate through the TCA cycle. These changes in the glutamate flux, and stability in metabolite incorporation capacity after GOT2 KD were corroborated, in principle, by the analysis of glutamine flux using ^13^C5, ^15^N2-glutamine (Fig. S4A, C).

The reduction of aspartate production from glutamate suggests that downstream metabolites of aspartate are possibly involved in the decreased glioblastoma cell growth upon GOT2 KD (Fig. 4D). Therefore, we semi-quantitatively compared the abundance of 30 metabolites, which are theoretically derived from glutamate, between shGOT2- and shScr-transduced cells. The changes in the abundance of metabolites were expressed as a ratio between shGOT2 and shScr. Twenty-eight metabolites were detected in at least three biological replicates of either shGOT2 or shScr. Of all metabolites detected, intermediate metabolites of nucleotide synthesis [34], that is, adenosine 5’-monophosphate (AMP), inosinic acid (IMP) and uridine 5’-monophosphate (UMP), showed the most significant reduction in their amount (Fig. 4E, Fig. S4D). These results might suggest that nucleotide synthesis is reduced upon GOT2 KD in glioblastoma cells.

Reduced nucleotide abundance upon GOT2 KD prompted us to hypothesize that GOT2 KD attenuates glioblastoma cell growth by suppressing DNA synthesis in the S-phase of the cell cycle. To address this hypothesis, we examined the degree of DNA synthesis in the S-phase by two methods: (i) DNA abundance measured by DAPI and (ii) incorporation of 5-Ethynyl Uridine (EdU), a thymidine analogue, into cells. Glioblastoma cells transduced with shGOT2 or shScr were incubated with EdU for 30 min prior to the fixation of the cells and then the DAPI and EdU intensities were analyzed by flow cytometry.

Based on the DAPI intensity, the proportion of cells in the S-phase was significantly reduced in glioblastoma cells with shGOT2 compared to those with shScr (Fig. 4F, G). Similarly, the proportion of glioblastoma cells that incorporated EdU was also decreased upon GOT2 KD (Fig. 4H, I).

Taken together, these results suggest that glioblastoma cells utilize GOT2-mediated evolutionarily adapted cell metabolism to provide nucleotide precursors, which are a prerequisite for glioblastoma cell cycle progression and thus growth.

## Discussion

The present study shows that an evolutionarily adapted cell metabolism, that is, glutaminolysis, is necessary for the growth of glioblastoma cells. We identified two key proteins in glutaminolysis, particularly in the conversion of glutamate to αKG, that play a crucial role in glioblastoma cell proliferation: one is human-specific mitochondrial glutaminolysis inducer ARHGAP11B, and the other is GOT2, a mitochondrial enzyme producing aspartate from glutamate. In the process of human evolution, human has acquired several human-specific genes, which have been shown to enhance human NSC’s proliferative capacity during fetal development [21, 54–57]. One of such genes is *ARHGAP11B*, whose protein is known to be localized in mitochondria to induce glutaminolysis [19]. As fetal development progresses, the expression of *ARHGAP11B* gradually decreases. Although the expression in the adult brain is very limited (Fig. 1A), we found relatively higher expression of ARHGAP11B in glioblastomas. The aberrant expression of ARHGAP11B in glioblastoma cells partly contributes to their malignant proliferative capacity, since inhibition of ARHGAP11B suppresses their proliferation. An increase in glutaminolysis is known to be a hallmark of highly mitotically active cells, especially tumor cells [58–60]. However, the mechanisms by which tumor cells upregulate glutaminolysis are largely unknown. The present study proposes a possible contribution of a human-specific gene to the increased glutaminolysis in tumor cells.

The last step of glutaminolysis, glutamate to αKG conversion, is mediated by three distinct types of enzymes [46, 60]. Of these enzymes, the role of GOT2 in tumor progression has been studied in non-brain tumors, but not in brain tumors [48, 61, 62]. On the one hand, GOT2 is shown to support pancreatic cancer growth [46, 62]. On the other hand, GOT2 acts as a tumor suppressor in hepatocellular carcinoma [62]. The present study shows that GOT2 expression is associated with the malignancy of glioblastoma. Although GOT2 is required for the growth of glioblastoma, it is not sufficient to promote cell growth. Therefore, GOT2 might constitute a prerequisite for glioblastoma cell growth. These previous and present studies suggest that the role of GOT2 is tumor type dependent. The reason why different tumor cells exhibit distinct dependence on the GOT2-mediated glutaminolysis remains elusive. One hypothesis is that differential expression of *GOT2* causes the distinct role of GOT2 in these tumor cells (Fig. S5). Another interesting hypothesis is that the differential expression of ARHGAP11B causes distinct activity of glutaminolysis, thus dependency on GOT2. This idea is corroborated by the distinct *ARHGAP11B* expression among different types of tumors. Of the above-mentioned three tumor types, glioblastoma shows the highest expression, followed by pancreatic adenocarcinoma, and hepatocellular carcinoma exhibits the lowest expression (Fig. S5). Whether ARHGAP11B is required for the growth of tumors other than glioblastoma needs to be experimentally addressed in the future.

Cell metabolism is a master regulator of cell growth because highly proliferating cells, such as tumor cells, utilize specific cell metabolism to support their astounding proliferative capacity [13–18]. Although previous studies showed the importance of ARHGAP11B-induced glutaminolysis in the high proliferative capacity of human NSCs [17, 19], the downstream mechanisms by which glutaminolysis promotes cell proliferation remain elusive. The present study indicates that the production of nucleotide precursors mediated by the GOT2-regulated glutaminolysis might constitute a prerequisite for high proliferative capacity of glioblastoma cells, thus their growth. Therefore, this pathway might be a good target for anti-glioblastoma therapy and novel diagnostic strategies.

## Conclusions

Human evolution gave us not only good features, but also deteriorative factors. Glioblastoma cells may hijack the molecular machinery of the higher proliferative capacity of human NSCs to support their aggressive growth. Our findings indicate that glioblastoma cells take advantage of an evolutionarily adapted metabolic program, originally supporting human fetal NSC expansion, to sustain malignant growth. Targeting ARHGAP11B-driven glutaminolysis mediated by GOT2 may offer a promising therapeutic strategy for glioblastoma.

### Limitation of the study

Our metabolomics was semi-quantitative, therefore, the data do not represent the absolute abundance of metabolites.

Although a decrease in aspartate and nucleotide precursor abundance, as well as a reduction in the proportion of cells in S phase, was experimentally demonstrated upon GOT2 knockdown (KD) (Fig. 4), the causal relationship between these metabolic changes and the GOT2 KD–induced growth inhibition of glioblastoma cells remains to be directly established. Further experimental studies will be required to determine whether the reduced availability of these metabolites is mechanistically responsible for the observed impairment in cell proliferation.

In addition, validation of ARHGAP11B and GOT2 protein expression was limited to patient‑derived glioblastoma cell lines. To enhance the translational relevance of the present findings, future studies should examine protein expression levels in glioblastoma tumor tissue samples obtained from patient biopsies and assess their correlation with clinical parameters, including patient prognosis.

## Supplementary Information

Below is the link to the electronic supplementary material.

Supplementary Material 1

Supplementary Material 1

## Data Availability

All data are available in the manuscript or the supplementary material, or publicly available as indicated in the Materials and Methods. Glioblastoma cells are available upon reasonable request to PL. Other materials are available upon reasonable request to TN.

## Abbreviations

αKG: Alpha-ketoglutarate
AMP: Adenosine 5’-monophosphate
bFGF: Basic fibroblast growth factor
BPTES: Bis-2-(5-phenylacetamido-1,3,4-thiadiazol-2-yl)ethyl sulfide
BSA: Bovine serum albumin
CGGA: Chinese glioma genome atlas
DAPI: 4’,6-diamidino-2-phenylindole
DN: Dominant negative
DNA: Deoxyribonucleic acid
EdU: 5-Ethynyl Uridine
EGF: Epidermal growth factor
G0/G1: Gap 0/Gap 1
G2/M: Gap 2/Mitosis
GBU: Genome Biology Unit
GFP: Green fluorescent protein
GLS1: Glutaminase 1
GLS2: Glutaminase 2
GLUD1: Glutamate dehydrogenase 1
GLUD2: Glutamate dehydrogenase 2
GPT1: glutamic-pyruvic transaminase 1
GPT2: glutamic-pyruvic transaminase 2
GOT1: Glutamic-oxaloacetic transaminase 2
GOT2: Glutamic-oxaloacetic transaminase 2
HelVi-BVC: Helsinki Virus Cores - Biomedicum Virus Core
HiLIFE: Helsinki Institute of Life Science
iGOT: Inhibitor of glutamic-oxaloacetic transaminases
IMP: inosinic acid
KD: knockdown
NSCs: Neural stem/progenitor cells
OAA: oxaloacetate
PBS: Phosphate buffered saline
PFA: Paraformaldehyde
S: Synthesis
shGOT2: short hairpin for glutamic-oxaloacetic transaminase 2
shScr: scramble short hairpin ribonucleic acid
shRNA: short hairpin ribonucleic acid
TCA: Tricarboxylic acid
UMP: Uridine 5’-monophosphate
WHO: World Health Organization
