# Supplementary material for: Glioblastoma cells utilize evolutionarily adapted cell metabolism to promote their malignant proliferation
Source: Acta Neuropathol Commun. 2026 May 13;14:146. doi: 10.1186/s40478-026-02318-7 (PMC13348691; doi:10.1186/s40478-026-02318-7)
Supplement: Supplementary file 2 — Supplementary Material 1 [file 40478_2026_2318_MOESM2_ESM.docx]

**Supplemental materials**


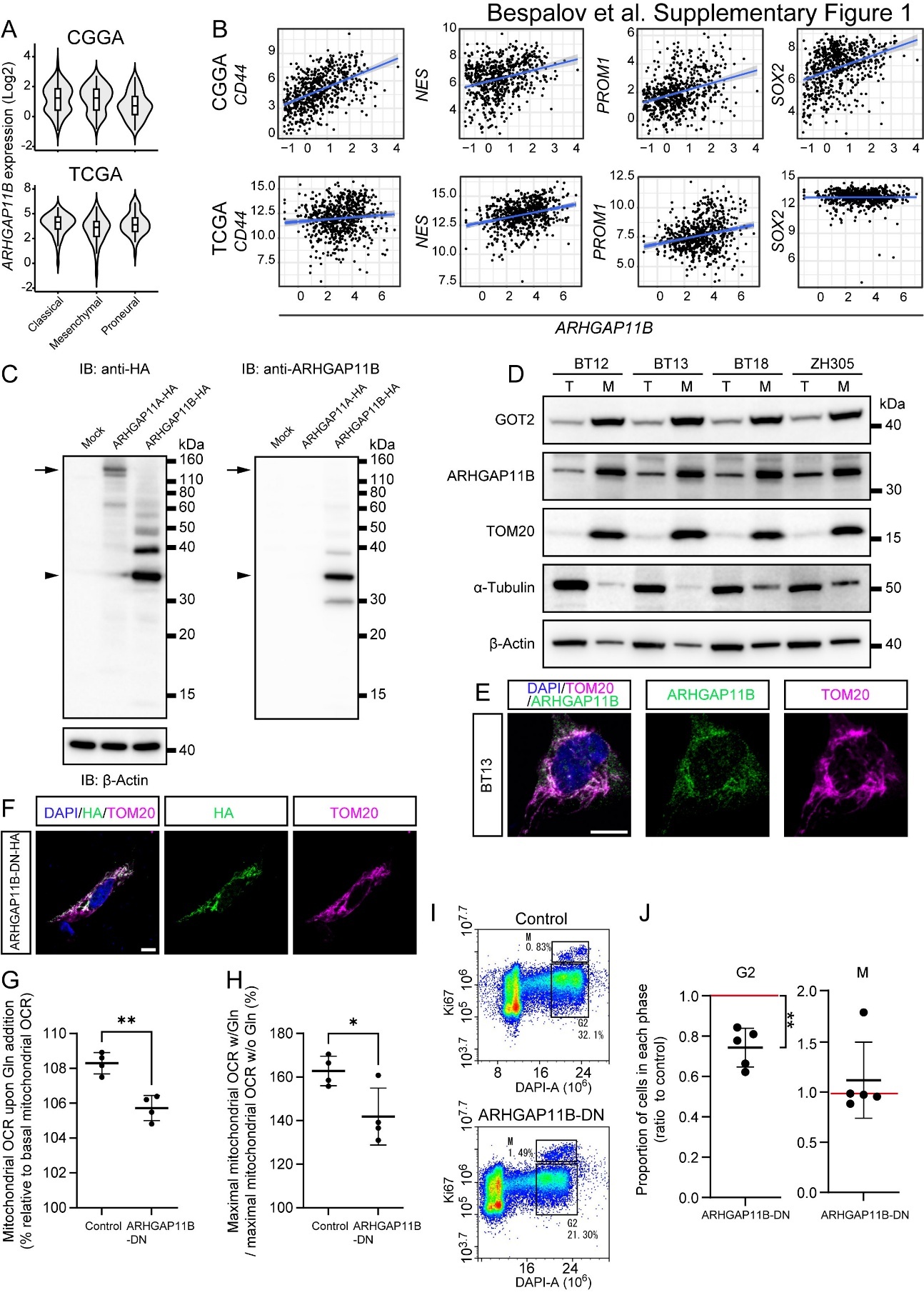
**Supplementary Figure 1: Expression of ARHGAP11B and its role in the cell cycle of glioblastoma cells.**

**A** Expression of *ARHGAP11B* in three glioblastoma subtypes: Classical, mesenchymal, proneural. Reanalysis of transcriptome data from CGGA (top panel) and TCGA (bottom panel) datasets. **B** Correlation of *ARHGAP11B* expression and gene expression of stem cell markers indicated in ordinates. Reanalysis of transcriptome data from CGGA (top panel) amd TCGA (bottom panel) datasets. **C** Validation of the newly generated rabbit anti-ARHGAP11B antibody by immunoblotting (IB). COS7 cell lysates after transfection of either pCAG-empty (Mock), pCAG-ARHGAP11A-HA or pCAG-ARHGAP11B-HA were subjected to immunoblotting using either the newly generated anti-ARHGAP11B antibody (right panel), anti-HA antibody (left top panel), or anti-beta-actin antibody as a loading control (left bottom panel). Arrows and arrowheads indicate ARHGAP11A and ARHGAP11B, respectively. D Total cell lysate (T) and mitochondrial fraction (M) were subjected to immunoblotting using either anti-GOT2, rabbit anti-ARHGAP11B, TOM20, anti-alpha-tubulin or anti-beta-actin. Note that all three known mitochondrial proteins (GOT2, ARHGAP11B and TOM20) are enriched in the mitochondrial fraction. **E** Immunofluorescence of a BT13 glioblastoma cell for TOM20 (magenta), ARHGAP11B (green; rabbit antibody) with DAPI staining (blue). Scale bar: 10 μm. **F** Immunofluorescence of a BT13 glioblastoma cell, which were transfected with pCAG-ARHGAP11B-DN, for TOM20 (magenta), HA (green) with DAPI staining (blue). Scale bar: 10 μm. **G, H** Mitochondrial oxygen consumption rate (OCR) measured by the Seahorse analyzer. BT13 cells were transduced with either GFP-expressing (Control) or dominant negative (ARHGAP11B-DN)-expressing lentivirus 2 days prior to analyses. **G** Mitochondrial OCR after the addition of 10 mM galactose and 2 mM glutamine (Gln) is expressed as a percentage of the mitochondrial OCR before the addition of the metabolites (named basal mitochondrial OCR). ***p* = 0.0017, *t* = 5.436, df=5.848, Welch’s t-test. **H** Percentage of maximal mitochondrial OCR in the presence of 2 μM of oligomycin, 100 μM of the uncoupler 2,4-dinitrophenol (DNP), 10 mM glucose, and 2 mM glutamine to the maximal mitochondrial OCR measured with oligomycin, DNP, and glucose but in the absence of glutamine. **p* = 0.0406, *t* = 2.840, df=4.525, Welch’s t-test. **I, J** Flow cytometry analysis of BT13 cells in the M and G2-phases (**I**: abscissa, DAPI area intensity (DAPI-A); ordinate, Cy3 height intensity for Ki67). Quantification of the proportion of cells in the G2- (left panel) and M- (right panel) phases of the cell cycle upon ARHGAP11B-DN overexpression (**J**). Statistical significance was determined by a one-sample t-test. ***p* = 0.0039, t = 5.991, df = 4 for G2, *p* = 0.5899, t = 0.5851, df = 4 for M.Error bars, SD.


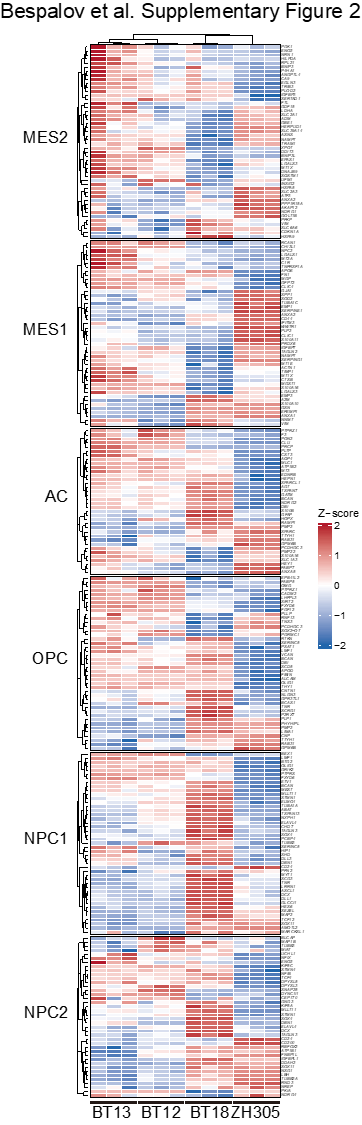


**Supplementary Figure 2: Characterization of glioblastoma cells.**

Characterization of glioblastoma cells by RNA sequencing. Clustered heatmap of BT12, BT13, BT18 and ZH305 cells based on glioblastoma subtype signature genes, which represent neural progenitor-like (NPC1 and NPC2), oligodendrocyte-progenitor-like (OPC), astrocyte-like (AC) and mesenchymal like (MES1 and MES2) [32]. Horizontal rows (genes) and vertical columns (replicates of each of the four cell lines) were hierarchically clustered. Note that each replicate from a given cell line were clustered primary, and BT12 cells and BT13 cells, and BT18 cells and ZH305 cells were clustered secondary, suggesting their similarities.


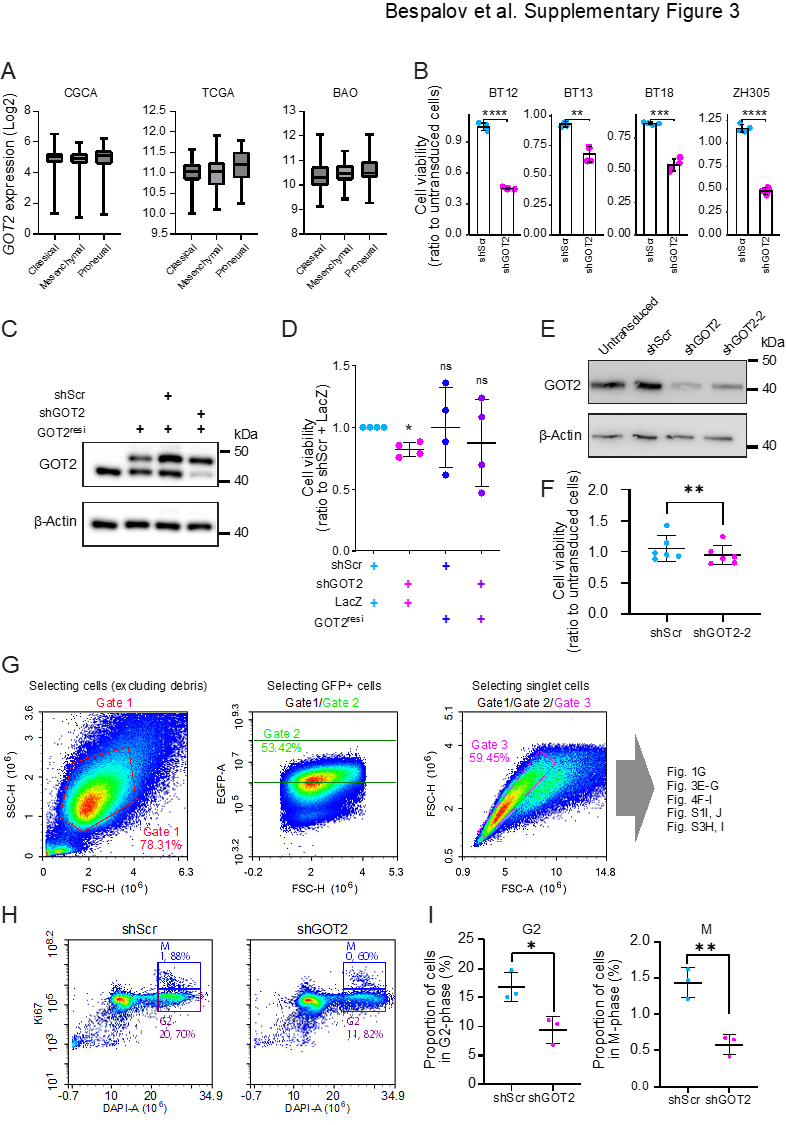


**Supplementary Figure 3: Effectsof GOT2 knockdown and sorting strategy of transduced cells.**

**A** Expression of *GOT2* in three glioblastoma subtypes: Classical, mesenchymal, proneural. Reanalysis of transcriptome data from CGGA (left panel) TCGA (middle panel) and Bao (right panel) datasets. **B** Quantification of cell viability of BT12 (left-most panel), BT13 (second left panel), BT18 (second right panel) and ZH305 (right-most panel) glioblastoma cells transduced with lentivirus carrying shScr (cyan) or shGOT2 (magenta) 7 days before the analysis. BT12: *****p* = 0.000041, t = 37.766, df = 3, BT13: ***p* = 0.0055, t = 7.196, df = 3, BT18: ****p* = 0.00068, t = 14.685, df = 3, ZH305: *****p* = 0.000071, t = 31.43, df = 3. **C** Expression of shGOT2-resistant GOT2 (GOT2^resi^) with or without shRNA against GOT2 (shGOT2) and shRNA with a scramble sequence (shScr) was examined by immunoblot using anti-GOT2 antibody. The upper bands and lower bands are exogenous and endogenous GOT2, respectively. β-actin was used as a loading control. **D** Quantification of cell viability of BT13 (left panel) glioblastoma cells transduced with the following combinations of lentivirus: shScr and LacZ (cyan), shGOT2 and LacZ (magenta), shScr and GOT2^resi^ (dark blue), shGOT2 and GOT2^resi^ (purple). The cell viability is expressed as a ratio of transduced cells to shScr- and LacZ-transduced cells. Statistical significance was determined by one sample t-test with Bonferroni correction (shScr+LacZ vs shGOT2+LacZ: **p* = 0.0083, *t* = 6.236, df=3; shScr+LacZ vs shScr+GOT2^resi^: *p* = 0.998, *t* = 0.0033, df=3; shScr+LacZ vs shGOT2+GOT2^resi^: *p* = 0.52, *t* = 0.725, df=3). ns, not significant. **E** Effects of shRNA against GOT2 (shGOT2 and shGOT2-2) and shScr on the expression of GOT2 were examined by immunoblot using anti-GOT2 antibody. β-actin was used as a loading control. **F** Quantification of cell viability of BT13 glioblastoma cells transduced with lentivirus carrying shScr (cyan), or shGOT2-2 (magenta). The cell viability is expressed as a ratio of transduced cells to untransduced cells. Statistical significance was determined by paired t-test (***p* = 0.0068, *t* = 4.429, df=5). **G** Gating strategy of flow cytometry shown in Fig. 1G, 3E-G, 4F-I, S1I, J, S3H, I. Gate 1 (red, left panel) for selecting cells and excluding debris, gate 2 (green, middle panel) for selecting GFP+ cells, and gate 3 (magenta, right panel) for selecting singlet cells. FSC-A, forward scatter area; FSC-H, forward scatter height; SSC-H, side scatter height. **H**, **I** Flow cytometry analysis of cells with indications of the cells in the M and G2-phases (**H**: abscissa, DAPI area intensity (DAPI-A); ordinate, Cy3 height intensity for Ki67). Quantification of the proportion of cells in the G2- (left panel) and M- (right panel) phases of the cell cycle upon transduced with lentivirus carrying shScr (cyan) or shGOT2 (magenta) (**I**). Statistical significance was determined by a paired t-test. **p* = 0.032, t = 5.490, df = 2 for G2, ***p* = 0.0047, t = 14.61, df = 2 for M. Error bars, SD.

**Supplementary Figure 4: Effects of GOT2 knockdown on the amount of metabolites.**

**A-C** BT13 cells were transduced by lentivirus carrying shScr (light blue) or shGOT2 (magenta), and were incubated with 0.5mM of ^13^C5, ^15^N1-glutamate (A, top panel and B; A bottom panel and C) for 2 hours, followed by metabolome analysis. Normalized peak areas of ^13^C5, ^15^N1-glutamate (A, top panel) and ^13^C5, ^15^N2-glutamine (B, bottom panel). Isotopologue peak areas of alpha-ketoglutarate (αKG), aspartate (Asp) and glutamate (Glu) normalized by ^13^C5, ^15^N1-glutamate (B) or ^3^C5, ^15^N2-glutamine (C) peak areas. Statistical significance was determined by Welch’s t-test. **D** BT13 cells were transduced by lentivirus carrying shScr (light blue) or shGOT2 (magenta), followed by metabolome analysis. Heatmap of 28 metabolites detected, each expressed as a natural-log-transformed ratio of shGOT2 transduced cells to shScr-transduced cells (E). Statistical significance was determined by Welch’s t-test, with FDR correction. **p* < 0.05, ***p* < 0.01, ****p* < 0.001, ***:*p* < 0.0001. Error bars, SD.

**Supplementary Figure 5: *GOT2* and *ARHGAP11B* expression in various tumor tissues and non-tumor tissues.**

*GOT2* (top panel) and *ARHGAP11B* (bottom panel) expression in non-tumor tissues (gray) and tissues with tumors indicated in the labels (magenta). Reanalysis of transcriptome data from TCGA [38] and GTEx [64] using GEPIA2 [65]. **p* < 0.05 and log_2_ fold change cutoff = 0.5. Error bars, SD.
